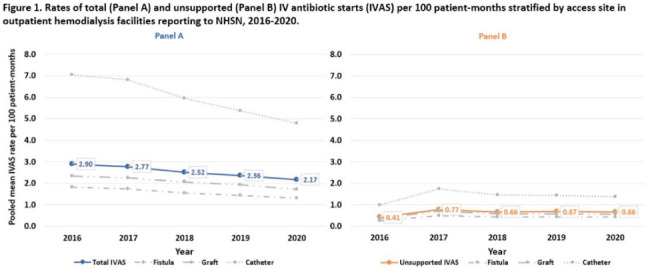# Rates of intravenous antibiotic starts among outpatient hemodialysis patients using NHSN dialysis event reporting, 2016–2020

**DOI:** 10.1017/ash.2022.194

**Published:** 2022-05-16

**Authors:** William Wilson, Sarah Kabbani, Shannon Novosad, Lucy Fike, Katryna Gouin, Jeneita Bell, Suparna Bagchi, Jonathan Edwards, Ibironke Apata, Susan Cali

## Abstract

**Background:** Nearly one-third of patients on hemodialysis receive intravenous (IV) antibiotics annually, but national data characterizing antibiotic use in this population are limited. Using NHSN surveillance data for outpatient dialysis facilities, we estimated temporal changes in the rate of IV antibiotic starts (IVAS) among hemodialysis patients as well as the proportion of IVAS that were not supported by a reported clinical indication. **Methods:** IVAS events were obtained from the NHSN Dialysis Event module between 2016 and 2020, excluding patients who were out of network, receiving peritoneal or home dialysis, or with unspecified vascular access. IVAS unsupported by documentation were defined as new IVAS without a collected or positive blood culture, pus, redness or swelling event, or an associated clinical symptom. Pooled mean rates of total and unsupported IVAS were estimated per 100 patient months yearly and stratified by vascular access type. Differences in IVAS rates by year were estimated with negative binomial regression. **Results:** Between 2016 and 2020, 7,278 facilities reported 648,410 IVAS events; 161,317 (25%) were unsupported by documentation (Table 1). In 2016, 3,340 (54%) facilities with ≥1 IVAS event reported an IVAS unsupported by documentation, which increased to 4,994 (73%) in 2020. Total IVAS rates decreased by an average of 8.2% annually (95% CI, 7.1%–9.3%; *P* < .001). The average annual percentage decrease did not differ significantly by vascular access site. The total IVAS rate was lowest in 2020 (2.17 per 100 patient months; 95% CI, 2.18–2.17). IVAS rates in 2020 were greatest for patients with catheter access (4.79 per 100 patient months; 95% CI, 4.75–4.83), followed by graft (1.71 per 100 patient months; 95% CI, 1.68–1.73), and lowest for patients with fistulas (1.30 per 100 patient months; 95% CI, 1.29–1.31). The overall pooled mean rate of unsupported IVAS was 0.64 per 100 patient months (95% CI, 0.63–0.64), which did not significantly change by year (Fig. 1). **Conclusions:** Total IVAS rates among outpatient hemodialysis patients have decreased since 2016, and rates among catheter patients remain highest compared to patients with fistulas or grafts. However, unsupported IVAS rates did not change, and the proportion of facilities reporting an unsupported IVAS increased annually. Targeted efforts to engage facilities with unsupported IVAS may help improve accurate reporting and prescribing practices.

**Funding:** None

**Disclosures:** None

**Table:**
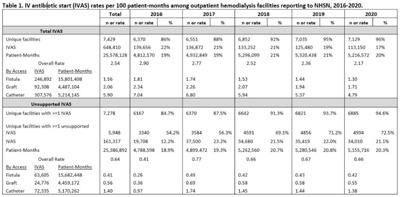

**Figure:**